# Impact of tailored feedback in assessment of communication skills for medical students

**DOI:** 10.3402/meo.v20.28453

**Published:** 2015-07-06

**Authors:** Seilin Uhm, Gui H. Lee, Jeong K. Jin, Yong I. Bak, Yeon O. Jeoung, Chan W. Kim

**Affiliations:** 1 Social Science Research Unit, UCL Institute of Education, University of London, London, UK; 2 School of Communication, Kookmin University, Seoul, South Korea; 3 Department of Medicine, Imperial College London, London, UK; 4 Department of German Language & Literature, Kongju National University, Gongju-si, South Korea; 5 German Studies Institute, Korea University, Seongbuk-gu, South Korea; 6 Department of Nursing, Kyung Dong University, Gangwon, South Korea; 7 Chung Ang University Hospital, Seoul, South Korea

**Keywords:** undergraduate medical training, communication, detailed feedback

## Abstract

**Background:**

Finding out the effective ways of teaching and assessing communication skills remain a challenging part of medication education. This study aims at exploring the usefulness and effectiveness of having additional feedback using qualitative analysis in assessment of communication skills in undergraduate medical training. We also determined the possibilities of using qualitative analysis in developing tailored strategies for improvement in communication skills training.

**Methods:**

This study was carried out on medical students (*n*=87) undergoing their final year clinical performance examination on communication skills using standardized patient by video-recording and transcribing their performances. Video-recordings of 26 students were randomly selected for qualitative analysis, and additional feedback was provided. We assessed the level of acceptance of communication skills scores between the study and nonstudy group and within the study group, before and after receiving feedback based on qualitative analysis.

**Results:**

There was a statistically significant increase in the level of acceptance of feedback after delivering additional feedback using qualitative analysis, where the percentage of agreement with feedback increased from 15.4 to 80.8% (*p*<0.001).

**Conclusions:**

Incorporating feedback based on qualitative analysis for communication skills assessment gives essential information for medical students to learn and self-reflect, which could potentially lead to improved communication skills. As evident from our study, feedback becomes more meaningful and effective with additional feedback using qualitative analysis.

Good communication skills of a doctor are central to delivery of high-quality medical care and have been shown to effect patient satisfaction and a variety of other biological, psychological, and social outcomes of patients (1–5).

Communication skills training has become an integral part of the undergraduate medical curriculum, where medical students are taught how to optimize the clinical effectiveness of communication skills for future clinical practice (6, 7). Therefore, communication skills must be assessed rigorously and objectively. Generally, Standardized Patient (SP) is the widely employed method for assessing communication and clinical skills (8). However, the assessment of communication skills can be subjective and has its inherent limitations (9). Furthermore, effective communication skills and their mechanism are difficult to investigate and measure (10).

Although there is ample evidence that incorporation of communication skills training in medical curriculum has been effective, little is known about the effective strategies for improving communication skills training (11, 12). For educators, there are variations in individual abilities in providing detailed and constructive feedback on the students’ communication skills (13, 14).

South Korea was the first country in Asia to introduce clinical skills examination as part of the medical licensing exams, where communication skills assessment was integrated into the Clinical Performance Examination (CPX) using SP (15). However, during the initial introductory phase of CPX, there was widespread criticism from the students on the lack of adequate communication skills training prior to CPX and there was disagreement with the scores given by SPs during CPX (16).

To enhance communication skills training in our institution, various feedback resources were offered to students aiming to deliberate practice-based learning after CPX. These included comments from SPs and opportunity to self-review the video-clips of their performance (5, 17–21). However, despite these measures, students had difficulty in identifying their strengths and limitations in communication skills. Medical students received insufficient guidance and feedback on their communication skills with the existing setup, which has been previously reported in other institutions (22, 23).

The aim of this study is to assess the role of additional feedback tool using qualitative analysis in communication skills assessment, which provides more detailed analysis of an individual student's verbal and nonverbal communication skills. Furthermore, we aim to develop a more effective communication skills training using qualitative analysis which studies the frequently observed behaviors of students during simulated consultations.

## Methods

### Clinical performance examination and assessment by standardized patient

In 2010, the final year students in our medical school underwent CPX consisting of six case scenarios under the auspices of Seoul-Gyeonggi CPX consortium.

Each student was given 12 min to assess one SP. After each encounter, the SP rated the student's performance using a checklist developed by the consortium. The checklist encompassed three areas: history taking, physical examination, and communication skills. The criteria for assessing communication skills consisted of the following seven sub-items: 1) the doctor made me feel comfortable/friendly; 2) the doctor listened to me sufficiently; 3) the doctor showed interest in my quality of life in addition to the health condition; 4) the doctor maintained a comfortable environment throughout the consultation; 5) the doctor treated me with respect and good manners; 6) the doctor's explanation was easy to understand; and 7) I felt confident with the doctor. The SP scored the above seven sub-items with a Likert scale of 0 to 5 points. Once this was completed, students were informed of their scores without any further reflective sessions.

Assessments were all video-recorded as part of our regular assessment, and informed consent was obtained prior to the assessment.

### Qualitative approach to understand students’ behavior in assessment

We built a team consisting of two linguists and two medical educators. Firstly, the team established qualities required for good communication skills based on existing six sub-items. We randomly selected 30 out of 87 video-clips for the assessment and 4 were excluded due to technical reasons.

The video-recordings were transcribed and analyzed under two main domains: verbal and nonverbal communication. We analyzed the verbal elements of communication using the transcripts, while the nonverbal elements were analyzed using the video-clips.

Once the linguists completed the assessment on communication skills, medical educators discussed the results with them in a case conference. A detailed feedback form was completed for each student highlighting the positive and negative points. In addition, we looked into the patterns of behaviors in students to fully evaluate their communication skills.

### Feedback to students

Two months after the examination, we delivered our routine feedback for all students (*n*=87), which included the scores and SP comments. All students had the opportunity to review the video-clips of their performance. We provided additional feedback using qualitative analysis to the randomly selected students in the study group (*n*=26). At the end of the session, we assessed the level of agreement of students with their scores on communication skills from both groups. Students also provided feedback on the effectiveness and usefulness of the additional feedback.

### Content analysis/thematic analysis

Contents within the personal feedback forms provided to the study group were divided into categories in the assessment tool depending on their intentions observed by researchers. They coded the categorized contents into specific skills, which students demonstrated to deliver the meaning. The coded skills were checked by the frequency of involvement and further categorized as either positive or negative point based on the transcript and overall context.

### Assessing the level of acceptance of scores after additional feedback based on qualitative analysis

The level of acceptance of scores was obtained using a Likert scale of five points: 1 (totally disagree) to 5 (totally agree). We further sorted the scale of five points into three categories: disagree, neutral, and agree.

Firstly, we analyzed the differences in the level of acceptance of scores between the control and study group after initial routine feedback session, without any additional feedback. We then determined the differences in the level of acceptance of scores between the control and study group, after receiving additional feedback based on qualitative analysis. Difference in the level of acceptance was analyzed using *χ* ^*2*^ *test* using SPSS Version 14.

Ethics approval was obtained from the Institutional Review Board (IRB), College of Medicine at Chung Ang University.

## Results

The level of acceptance of scores between the control and study group showed no significant difference after the initial routine feedback session. However, there was a significant increase in the level of acceptance of scores in the study group after receiving additional feedback, increasing from 15.4 to 80.8% (see Table 1).

**Table 1:** Change in acceptance of feedback by adding information based on qualitative analysis

	After routine feedback*		After additional feedback
	
	Nonstudy group	Study group	Study group
Disagree	32 (52.4%)	13 (50.0%)	0 (0.0%)
Neutral	21 (34.4%)	9 (34.6%)	5 (19.2%)
Agree	8 (13.1%)	4 (15.4%)	21 (80.8%)**

* *p*=0.956: comparison of study group and nonstudy group within after routine feedback.

** *p*<0.001: comparison of after routine feedback and after feedback using qualitative analysis for study group (*N*=26).

### Students’ response after routine feedback

After our routine feedback following CPX, there was general consensus amongst students that they were dissatisfied with the feedback provided, where 45 out of 87 students disagreed with their communication skills scores. Disagreement was largely due to lack of appropriate explanation for their scores. Although we trained SPs to provide detailed comments to every student, due to time constraints during the examination, it was not always possible to provide a detailed feedback. As a result, some students had no feedback on their strengths and weaknesses, which was cordially raised afterwards.

### Students’ response after additional feedback using qualitative analysis

Students from the study group who received additional feedback with detailed analysis of their verbal and nonverbal communication were overall satisfied with the intervention. They commented that it was easier to identify the specific areas of strengths and weaknesses in communication skills. Students were able to replay the video-clips with guided commentaries on their performance. Nobody in the study group disagreed with their communication skills scores after intervention.

### Benefits for students with good scores

We have found out that the additional feedback was helpful for students with good scores as well. This group had received high scores in communication skills in the past but could not identify exactly how and why they performed well. Additional feedback provided insight into their qualities in demonstrating effective communication and enabled to build and maintain their strengths in communication skills.

### Frequently observed behaviors

Students showed similar behaviors which were frequently observed (see Table 2):

1. ‘Smiling and eye contact’: Smiles and good eye contact softened the mood of consultation and made SPs feel more comfortable. However, in some instances, students smiled looking elsewhere (e.g., the desk in front).
2. ‘Was it easy to find the hospital?’: When this comment was used at the beginning of the consultation, it made the SP comfortable and set the scene for the examination. It was perceived as a courteous and caring gesture of the student towards SP. However, in some cases, this comment was made in the middle of the consultation.
3. ‘How does it affect your daily activities?’: Several students successfully asked the effect of disease on the daily activities of the SP. This made the SP feel that the student was not just interested in the disease or health condition, but also in SP's quality of life. However, some students asked this question out of context, making SPs feel that the student was intruding on their private matters.
4. ‘It must be very difficult for you’: This expression was frequently used as a response to SP's clinical history and difficulties. When such a comment was used appropriately, SP felt that the student was empathic to their symptoms. However, in Korean language, this sentence could be misinterpreted as ‘you appear to be physically suffering’. Therefore, when this comment was made abruptly out of context, the SP felt patronized, especially when it was used inappropriately (e.g., one SP thought the student was commenting on her physical appearance).
5. ‘Everything will be fine’: This expression was commonly used when students attempted to reassure SPs showing signs of anxiety. There were instances where SPs felt reassured with this comment, but in some cases, students’ verbal and facial expressions were not corresponding.
6. ‘Paraphrasing and summarizing’: It was observed from several students. This was a good opportunity for students to check their understanding and confirm the history from SPs. However, when this was used too frequently or inappropriately, SPs felt that the students were not paying attention hence asking the SP to repeat.
7. ‘Respectful posture’ (sitting upright): During consultation, most students sat upright. Maintaining such posture throughout consultation is perceived as being respectful in Korean culture. We advise our students to avoid certain disrespectful behaviors such as crossing their legs, playing with their pens, and not keeping still. However, some students appeared too rigid and the SPs felt uncomfortable during consultation.
8. ‘Pause’: A period of silence during consultation gave appropriate time for the SP to speak and recuperate. However, prolonged silence made the SP feel uncomfortable and even anxious, wondering whether the SP had said something wrong.

**Table 2:** Frequently observed behaviors of students during interview and corresponding feedback

Observed behaviors (*n*=number of students)	Positive feedback	Negative feedback
Smiling at the SPs (*n*=22)	This could portray your kindness and warmth when demonstrated at the beginning of consultation	Smiling at SP who was describing their pain could be insulting
‘Was it easy to find the hospital?’ (*n*=19)	It could show your familiarity and concern for SP	When such comment is made in the middle of the conversation and out of context, it would give an impression that you had suddenly remembered to ask this
‘Does it affect your activities?’ (*n*=23)	Could show your concern about SP's quality of life beyond the disease itself	When said at the beginning right after introducing yourself, this could be inappropriate to SP
‘It must have been very difficult for you’ (*n*=18)	Shows sympathy towards the suffering SP	Without eye contact and proper posture, it could deliver the wrong message ‘I got angry … because it sounded like he was judging on my physical appearance’
‘Everything will be fine’ (*n*=21)	Good to sound caring, which could calm the SP	Communicating with cold and rigid look on the face could give an impression that you do not care about the SPs
Use of summary (*n*=21)	Good to check patients’ understanding	When used too frequently, it could imply that SP is unable to understand the explanation ‘I felt unpleasant to be considered stupid’
Sitting upright on the chair (*n*=24)	Good to appear polite, gentle, and respectful to the SP	Too rigid might come across as artificial. If needed turn lightly ‘I didn't like him looking like nova's posture of sitting upright continuously’, said SP
Pause (*n*=16)	Makes SP feel comfortable, enabling them to feel free to talk	Without proper intervals, you could appear to be hesitating ‘Silence was too long, so I began to be anxious thinking what's wrong with my talk and she looked clumsy’

## Discussion

This study highlighted the need for improvement in delivering the feedback for communication skills examination to both students and educators in the following areas.

### Enhance self-reflection through practice-based learning

Improving communication skills in terms of ‘practice-based learning’ involves multi-dimensional qualities: students should have personal insight into their performance (11), they should be able to learn from their encounters (24), and should be able to make changes in behavior based on self-reflection (25). Therefore, the ability to reflect on their behavior is essential in improving their communication skills. However, it is questionable whether students’ self-assessment would be reliable or accurate (26–28). The importance of providing additional feedback or educational intervention to improve self-assessment and reflection has been previously highlighted (26, 29).

Srinivasan et al. (26) studied the students’ self-assessment of their video-clips from CPX. It suggested significant improvement when additional information was provided. Martin et al. (29) argued that doctors’ self-evaluation skills improved when they watched and compared different levels of performance of their peers on video-clips. Similarly, we also observed that when students compared each other's video-clips, there was some improvement in their understanding of effective communication skills, but only to some extent. In students lacking the ability to self-reflect, this method was insufficient to provide adequate feedback and the intention to improve on communication skills. More concrete accounts addressing their own verbal or nonverbal cues were required.

Therefore, providing detailed feedback on students’ own behavior with straightforward references (such as time of video-clips or line/page numbers of transcripts) could be helpful to understand their strengths and weaknesses effectively. For example, one student described, ‘Previously, my results were just numbers so I had to guess my weaknesses. But with the detailed feedback, I can understand what my weaknesses are because they were clearly explained’ (Student 1).

### Understanding the overall ‘context’

We observed that students understood the overall ‘context’ of doctor–patient communication as they received additional feedback and self-reflected. From analyzing the frequently observed behaviors and student feedback, we identified the following elements, which resulted in poor communication despite students’ good intentions: 1) nonverbal languages, 2) eye-contact, and 3) timing.

When nonverbal language did not correspond to the verbal language, it delivered completely different social meaning to patients. ‘I thought I listened well, maintaining good manners and was courteous. However, from the feedback, I realized that my facial expression was different to what I said and realized that it could give a completely different impression’ (Student 2).

Another common realization from the students was the importance of eye contact. Reassuring words without appropriate eye contact could lead to patient feeling that the student did not care much, but was just ‘saying’ it. ‘I thought telling the SP that “everything was all right” would be reassuring. However, I realized that I could give a wrong impression despite saying the right thing, which was not my intention’ (Student 3).

Timing is also an important cue in understanding the context of communication. Teaching manuals often highlight ‘key’ words or phrases that aid to a comfortable consultation. However, there is a tendency for students to mechanically memorize these phrases and use it inappropriately, leading to breakdown in patient rapport and communication. In our study, this was a common observation in students that such ‘key’ phrases were used at the incorrect moment during the consultation. For example, a student thought that he/she was being polite by saying, ‘Was it easy to find the hospital?’ However, this question was used at the middle of consultation rather than during the initial stage and the SP felt confused and found it out of context.

The feedback based on qualitative analysis suggested that having a natural smile, appropriate eye-contact/posture, interim summarization, and active listening were important cues to make SPs feel more comfortable. Our students initially felt that they had adhered to the suggested behaviors. However, after receiving detailed feedback, they realized that their good intentions were not represented effectively: they smiled but it was unnatural, they tried to maintain eye contact but it was inappropriate, students remembered to summarize but it was too frequent, and they tried to listen to the patients, but the gap between the conversations was too long.

### Implication to medical educators

Common mistakes demonstrated by students could be associated with the existing method of communication skills training, where emphasis is made on didactic teaching on the theoretical principles of communication skills. As a result, students memorized the list of good communication principles and attempted to include all theories into the consultation, mostly out of context. Effective communication requires the ability to adapt, to be responsive, and to manage self-awareness during the process of talking and listening. Applying a random list of good communication cues did not work for some students, especially when they used the cues mechanically. Such undesirable patterns could be reflective of ineffective communication skills training, particularly in the Far East, where the importance of communication skills can often be underestimated.

### Suggested areas of improvement

Despite our study demonstrating the usefulness of providing additional feedback using qualitative analysis, it would be unrealistic to adopt this method in every CPX. It is time consuming and expensive. However, it is potentially useful in identifying the strengths and weaknesses in communication skills in a small cohort of students and in investigating the potential causes.

We identified limitations with the current setup of providing routine feedback using SPs. SPs must be adequately trained and assisted to provide appropriate level of feedback. Our study demonstrated that students benefited from detailed feedback, which was objective and consistent. For students who received no remarks due to time constraints on SPs, there was no educational benefit. Therefore, enough time should be allocated for SPs to give formative feedback to students. Additionally, there was a tendency for SPs to become subjective depending on students’ remarks (e.g., a student who complemented SP by saying she looked young for her age received good scores). This could be resolved by providing further training for SPs using examples from our study. Currently, SPs solely provide scores and feedback on communication skills. Therefore, another way to prevent subjectivity would be to have a second opinion, preferably a clinician. However, it remains a challenge in recruiting and training clinicians to be involved in CPX and providing adequate feedback on communication skills.

Most Korean medical schools follow the traditional curricula, where there is limited patient contact during their 6 years of undergraduate training. Students have their first patient contact during their clerkship period in their final year. Theory-based teaching is most commonly adopted. However, good communication skills cannot be effectively taught in lecture theatres. Students require exposure to more practical sessions in both simulated and clinical settings. Introducing patient contact at an earlier stage of undergraduate medical training will be beneficial for students, providing sufficient opportunity to communicate with real-patients. However, students should also have adequate prior training in a simulated environment with SPs (30).

### Limitations

This study has its inherent limitations. Small sample size, lack of randomization, and blinding could all potentially contribute to bias in the results. Our study was also conducted in an examination setting using SPs, which may not be representative of real-life clinical setting. There is also potential influence of having the input of clinicians and communication specialists in the study group, especially in a culture that values obedience and respect for educators.

## Conclusions

Our study demonstrated that providing students with additional feedback using qualitative analysis is beneficial for both students and educators. More students were able to agree with their scores and, most importantly, received adequate tool to improve on their communication skills through self-reflection. Equally for educators, qualitative analysis highlighted the common mistakes made by students in communication skills assessment. This study further discussed the need for increased participation and detailed feedback from SP and medical educators.

## References

[1] Simpson M, Buckman R, Stewart M, Maguire P, Lipkin M, Novack D (1991). Doctor–patient communication: the Toronto consensus statement. BMJ.

[2] World Federation for Medical Education (1994). World Summit on Medical Education. Proceedings. Edinburgh, 8–12 August 1993. Med Educ.

[3] Kurtz S, Silverman J, Benson J, Draper J (2003). Marrying content and process in clinical method teaching: enhancing the Calgary–Cambridge guides. Acad Med.

[4] Ruiz-Moral R, Perez Rodriguez E, Perula de Torres LA, de la Torre J (2006). Physician–patient communication: a study on the observed behaviours of specialty physicians and the ways their patients perceive them. Patient Educ Couns.

[5] Zick A, Granieri M, Makoul G (2007). First-year medical students’ assessment of their own communication skills: a video-based, open-ended approach. Patient Educ Couns.

[6] Hauer KE, Boscardin C, Gesundheit N, Nevins A, Srinivasan M, Fernandez A (2010). Impact of student ethnicity and patient-centredness on communication skills performance. Med Educ.

[7] Lumma-Sellenthin A (2009). Talking with patients and peers: medical students’ difficulties with learning communication skills. Med Teach.

[8] Hauer KE, Hodgson CS, Kerr KM, Teherani A, Irby DM (2005). A national study of medical student clinical skills assessment. Acad Med.

[9] Huntley CD, Salmon P, Fisher PL, Fletcher I, Young B (2012). LUCAS: a theoretically informed instrument to assess clinical communication in objective structured clinical examinations. Med Educ.

[10] Drew P, Chatwin J, Collins S (2001). Conversation analysis: a method for research into interactions between patients and health-care professionals. Health Expect.

[11] Noble LM, Kubacki A, Martin J, Lloyd M (2007). The effect of professional skills training on patient-centredness and confidence in communicating with patients. Med Educ.

[12] Martin J, Lloyd M, Singh S (2002). Professional attitudes: can they be taught and assessed in medical education?. Clin Med.

[13] Chang A, Boscardin C, Chou CL, Loeser H, Hauer KE (2009). Predicting failing performance on a standardized patient clinical performance examination: the importance of communication and professionalism skills deficits. Acad Med.

[14] Dudek NL, Marks MB, Regehr G (2005). Failure to fail: the perspectives of clinical supervisors. Acad Med.

[15] Kim KS (2010). Introduction and administration of the clinical skill test of the medical licensing examination, republic of Korea (2009). J Educ Eval Health Prof.

[16] Huh S (2010). Failed examinees’ legal challenge over the clinical skill test in the Korean Medical Licensing Examination. J Educ Eval Health Prof.

[17] Ward M, MacRae H, Schlachta C, Mamazza J, Poulin E, Reznick R (2003). Resident self-assessment of operative performance. Am J Surg.

[18] Epstein RM (1999). Mindful practice. JAMA.

[19] Hays RB, Jolly BC, Caldon LJ, McCrorie P, McAvoy PA, McManus IC (2002). Is insight important? Measuring capacity to change performance. Med Educ.

[20] Gordon MJ (1992). Self-assessment programs and their implications for health professions training. Acad Med.

[21] Langendyk V (2006). Not knowing that they do not know: self-assessment accuracy of third-year medical students. Med Educ.

[22] Howley LD, Wilson WG (2004). Direct observation of students during clerkship rotations: a multiyear descriptive study. Acad Med.

[23] Colletti LM (2000). Difficulty with negative feedback: face-to-face evaluation of junior medical student clinical performance results in grade inflation. J Surg Res.

[24] Borrell-Carrio F, Epstein RM (2004). Preventing errors in clinical practice: a call for self-awareness. Ann Fam Med.

[25] Ziegelstein RC, Fiebach NH (2004). ‘The mirror’ and ‘the village’: a new method for teaching practice-based learning and improvement and systems-based practice. Acad Med.

[26] Srinivasan M, Hauer KE, Der-Martirosian C, Wilkes M, Gesundheit N (2007). Does feedback matter? Practice-based learning for medical students after a multi-institutional clinical performance examination. Med Educ.

[27] Davis DA, Mazmanian PE, Fordis M, Van Harrison R, Thorpe KE, Perrier L (2006). Accuracy of physician self-assessment compared with observed measures of competence: a systematic review. JAMA.

[28] Wood J, Collins J, Burnside ES, Albanese MA, Propeck PA, Kelcz F (2004). Patient, faculty, and self-assessment of radiology resident performance: a 360-degree method of measuring professionalism and interpersonal/communication skills. Acad Radiol.

[29] Martin D, Regehr G, Hodges B, McNaughton N (1998). Using video-taped benchmarks to improve the self-assessment ability of family practice residents. Acad Med.

[30] Schaufelberger M, Frey P, Woermann U, Schnabel K, Barth J (2012). Benefits of communication skills training after real patient exposure. Clin Teach.

